# Physical Properties of Substrates as a Driver for *Hermetia illucens* (L.) (Diptera: Stratiomyidae) Larvae Growth

**DOI:** 10.3390/insects14030266

**Published:** 2023-03-08

**Authors:** Wael Yakti, Marcus Müller, Martina Klost, Inga Mewis, Dennis Dannehl, Christian Ulrichs

**Affiliations:** 1 Urban Plant Ecophysiology Division, Faculty of Life Sciences, Thaer-Institute of Agricultural and Horticultural Sciences, Humboldt-Universität zu Berlin, Lentzeallee 55, 14195 Berlin, Germany; 2 Department of Food Technology and Food Material Science, Institute for Food Technology and Food Chemistry, Faculty III Process Sciences, Technische Universität Berlin, Straße des 17. Juni 135, 10623 Berlin, Germany; 3 Division Biosystems Engineering, Faculty of Life Sciences, Thaer-Institute of Agricultural and Horticultural Sciences, Humboldt-Universität zu Berlin, Albrecht-Thaer-Weg 3, 14195 Berlin, Germany

**Keywords:** black soldier fly, physical properties, fibres, insect protein

## Abstract

**Simple Summary:**

This study aimed to investigate how the growth and nutrient composition of black soldier fly larvae could differ based on the physical structure of the feed given to the larvae. The results show that manipulating only the physical properties of the substrate can change larval growth, survival rate, and body composition. These results can help optimise the substrate by modifying its physical properties.

**Abstract:**

The growth and nutritional profile of the black soldier fly larvae (BSFL) is usually investigated and compared when the larvae feed on substrates that differ in the chemical composition as well as physical properties. This study compares BSFL growth on substrates that differ primarily in physical properties. This was achieved by using various fibres in the substrates. In the first experiment, two substrates with 20% or 14% chicken feed were mixed with three fibres (cellulose, lignocellulose, or straw). In the second experiment, the growth of BSFL was compared with a 17% chicken feed substrate that additionally contained straw with different particle sizes. We show that the substrate texture properties values did not influence the BSFL growth, but the bulk density of the fibre component did. The substrate mixed with cellulose led to higher larvae growth over time in comparison to substrates with higher bulk density fibres. BSFL grown on the substrate mixed with cellulose reached their maximum weight in 6 days instead of 7. Neither the fibres nor the nutrient level changed the crude protein content of BSFL and the values ranged between 33.5% and 38.3%, but an interaction between the fibre and nutrient level was observed. The size of straw particles in the substrates influenced the BSFL growth and led to a 26.78% difference in Ca concentration, a 12.04% difference in Mg concentration, and a 35.34% difference in P concentration. Our findings indicate that the BSFL-rearing substrates can be optimised by changing the fibre component or its particle size. This can improve the survival rate, reduce the cultivation time needed to reach the maximum weight, and alter the chemical composition of BSFL.

## 1. Introduction

Protein-rich ingredients are considered the most crucial and expensive feed component. The production of soybean, for example, exploded during recent decades, reaching more than 353 million tons in 2020, and needed over 126 million hectares of agricultural land [1]. Growing human-edible crops such as soybean and grains for the production of animal feed is a matter of controversy as it puts feed production in direct competition with human food for agricultural land [2]. Additionally, the current feed-protein supply cannot keep up with the expanding animal production. It is, therefore, necessary to explore new sources of proteins for food and/or feed which can be produced with less land use [3]. Insect farming has emerged as an expanding approach for the production of high-quality proteins from low-value waste streams and in limited spaces, which contributes to global food security [4]. Additionally, when provided with suitable diets, insects can utilise proteins at a higher efficiency than conventional livestock [5].

The black soldier fly (BSF), *Hermetia illucens* (Diptera: Stratiomyidae), is a saprophagous insect that can grow on organic wastes, accumulates high-quality proteins, and can be used as animal feed [6,7]. The larvae (BSFL) feed on a wide spectrum of substrates, including manure [7,8] and food wastes [9], and their implementation in waste management can reduce greenhouse gas emissions [10]. In addition to proteins, fats can be extracted from BSFL biomass and used as a feed ingredient [11], in cosmetics [12], or to produce biofuel [13]. BSFL excrement, also known as frass, can be used as a plant fertiliser [14]. The adoption of the BSF bioconversion process fits in circular economies and can contribute to meeting the ever-increasing demand for animal feed [15].

The growing interest in BSF has led to the accumulation of studies that investigate factors influencing its growth including temperature [16], larvae density and batch size [17,18], and substrate moisture [19]. The vast majority of studies assessed the performance and composition of BSFL grown on different substrates and waste streams [20]. When different substrates are compared in experimental setups, they often differ not only in their nutritional value but also in their physical properties. The influences of substrates’ physical parameters such as viscosity, texture, and porosity have not been adequately investigated even though they could impact BSFL growth regardless of the nutritional value of the substrate. In a study conducted by Palma et al. [21], an increased almond hull particle size increased larval mass by 10%, which could be attributed to substrate texture and aeration. These factors are rarely discussed in BSF literature. Adding a “solid support” component has been shown to improve the performance of BSFL fed with liquid leachate [22]. In a further study by Grossule et al. [23], different fibre-rich components such as wheat bran and brewers’ spent grain were used as a “solid support”, but in this case, the differences in BSFL growth could be attributed to both the “physical support” and the added nutritional value of these additives. Fibrous components with high porosity may increase aeration in high-moisture substrates. This could directly influence BSFL growth and microbial activity which can reflect on the growth of larvae [24,25].

Excess water and other liquids in the substrate such as fats potentially reduce the growth of larvae, hinder their feeding and free movement, and increase mortality [26,27,28,29]. Although adding fibres could improve the physical structure of the substrate, they are not readily digestible as a nutrient for BSFL, leading to a negative impact on the bioconversion process if they are to replace a nutritive component [30]. It is, therefore, important to identify the desired physical characteristics of substrates for optimal BSFL growth. If these physical substrate properties are known, BSFL-based conversion processes can be optimised at low cost, e.g., by amending growth substrates with low-value fibres and reaching the appropriate texture. Fibres with a sufficient water-holding capacity could prevent the separation of liquids from the solid components in the feed. Additionally, low-bulk density fibres could allow higher pore volume, better aeration, and subsequently a better substrate matrix that could support the feeding, movement, and gas exchange needed for BSFL growth.

The current study seeks to explore the influence of substrates’ physical properties on the growth of BSFL. The physical properties of the substrates were altered by adding different fibre components or by adding straw with different particle sizes to the substrates. We hypothesised a relation between substrates’ physical properties (texture, bulk density, or water holding capacity) and BSFL growth. This understanding can support substrate optimisation efforts by BSF producers and can help researchers to formulate diets for studying BSFL nutrition whilst minimising the bias that is potentially introduced when substrates with different physical properties are used.

## 2. Materials and Methods

### 2.1. Black Soldier Fly Colony

The BSF colony used in this study was obtained from the Leibniz-Institute of Freshwater Ecology and Inland Fisheries (IGB) (Berlin, Germany) and was maintained in a rearing hall in Dahlewitz (Blankenfelde-Mahlow, Germany). The neonates used in the experiments were reared as described in Yakti et al. [18,31] and received a chicken feed diet (Product name and batch: K (11 4) o.K., Agravis Raiffeisen AG, Velten, Germany).

### 2.2. Experiment 1: The Influence of Different Fibre Components and Nutrient Levels on BSFL Growth

A factorial experiment was carried out to investigate the influence of different substrates’ physical properties on the growth of BSFL under two different nutritional levels. Three different fibre types were each mixed with two different ratios of chicken feed as the nutritive component (Table 1). The fibre types used were: (1) powder cellulose (ARBOCEL^®^ BC 1000, Rettenmaier & Söhne GmbH + Co KG, Rosenberg, Germany), (2) concentrated lignocellulose (ARBOCEL^®^ EFC 1350, Rettenmaier & Söhne GmbH + Co KG, Rosenberg, Germany), or (3) blended straw (Futtermittel Kuhnwald GmbH, Friedland, Deutschland). The substrates as fed contained 26.83% dry matter and were mixed with water using a kitchen spatula, and then were incubated at room temperature for at least four hours to allow the fibres to soak in water. The substrates were mixed again before adding the insects. For each mixture, 5 replicates of 500 g were produced with the physical properties shown in Table 1. To conserve the dry matter content of the rearing substrates with different quantities of chicken feed, the reduced weight was compensated by blended straw. Polyethylene boxes with an area of 12 × 17 cm were filled with 500 g substrate, and 500 larvae (7 days old with an average weight of 3.6 mg) were added on the top for each box. The boxes were unlidded and maintained at 30 °C and 30–40% rH for 6 or 7 days. Every day, 50 to 100 larvae were collected from every box, weighted, and returned to the substrate. The larvae were fully harvested when a decreasing trend in the average larvae weight was observed.

A subsample of 100 mL was used to determine the bulk densities of the wet substrate mixtures (WetBDens). The determination of the WetBDens was performed by dividing the weight of the material by the volume occupied. To measure the water holding capacities (WatHC) of the different fibres used in the experiment, a weighed dry amount of each fibre (DryWt) was added to a 150 mL cylinder with water-permeable bottoms. The DryWt was noted, allowing the calculation of the DryBDens. The fibre was saturated by a capillary rise in a water bath for 24 h. Thereafter, the cylinders’ openings were covered with parafilm to exclude evaporation, and excess water was allowed to drain for 24 h. Dividing the weight of the water-saturated fibre (WetWt) or the DryWt by 0.15 (the cylinder volume in litres) allows the calculation of the WetBDens or DryBDens of fibres. The water holding capacity of the fibres (WatHC) was calculated as follows:

$$\mathrm{WatHC}\;\left({\mathrm{mL}\;g}^{-1}\right)=\frac{\left(\mathrm{WetWt}-\mathrm{DryWt}\right)}{\mathrm{DryWt}}\;$$

#### Texture Measurements

The substrate mixes were subjected to a penetration test to assess the texture. The force required to penetrate the samples was measured using a Zwick/0.5 materials testing machine (ZwickRoell GmbH & Co. KG, Ulm, Germany). Three aliquots per substrate were filled into beakers (43 mm Ø and 60 mm high), and the force required to penetrate each of these aliquots for a distance of 15 mm was measured with a cylindrical probe (10 mm Ø) at a speed of 2 mm s^−1^.

The linear regression to the force–distance data resulted in R^2^ values of at least 0.9911 (feed LN1, Table 2) and the differences were mainly in the slopes. Therefore, the force required to penetrate the substrate differed with the components of the substrates.

### 2.3. Experiment 2: The Influence of Different Straw Particle Sizes on BSFL Growth

In this experiment, the physical properties of the substrates were altered by adding straw with different particle sizes. The straw (Futtermittel Kuhnwald GmbH, Friedland, Deutschland) was blended and then sieved to obtain 5 straw fractions with different particle sizes (see Table 3). These fractions were mixed to produce substrates to a ratio of 17% chicken feed, 5.5% straw, and 77.5% water (weight based). The amount of chicken feed was set to provide a nutritional value laying between the two levels tested in experiment 1. The straw fractions and their properties are shown in Table 3.

The substrates and rearing boxes were prepared as in experiment 1, and the experiment was carried out also in five replicates. Each box had 500 g of substrate and 425 larvae (7 days old with an average weight of 6 mg). The larvae grew for 6 or 7 days and, as in the first experiment, were harvested when the weight decrease in the weight of single larvae was recorded.

### 2.4. Determination of Growth and Biomass Parameters, and Chemical Analysis

In both experiments, the larvae were sampled by collecting and weighing 50–100 larvae per box on a daily basis. At the time of harvest, all the larvae were separated from the rest substrate at room temperature and were counted manually to determine survival rates. The total weight of the larvae and the weight of the rest substrates were measured. The larvae and the rest substrates were dried overnight at 60 °C in an oven, and the dry weight was determined after confirming that no further weight reduction took place. Additionally, the feed conversion ratio (FCR) was estimated by dividing the reduction in dry substrate weight by the total dry weight gain of the larvae.

For the chemical analysis of P, N, Ca, Mg, K, and Fe, oven-dried larvae were grounded and analysed as described in Yakti et al. [18]. All analyses were conducted in three biological and two technical replicates.

### 2.5. Statistical Analyses

A linear mixed-effect model with maximum likelihood estimation was used to analyse the effects of the factors on the growth of larvae over time (repeated measures) in both experiments. An unstructured “correlation metric” covariance structure was used in the model to avoid assumptions related to correlation homogeneity between the measurements considering that the growth of larvae increased and then decreased before harvest. The linear mixed-effect model analysis was followed by a subsequent Bonferroni test to uncover the specific differences between the groups. For the rest of the parameters that did not have repeated measures, ANOVA or two-way ANOVA was used. Additionally, a Bonferroni post hoc test was used after the ANOVA for pair-wise comparisons. The data were tested for normality and homogeneity of variance before ANOVA, and a Kruskal–Wallis test was used when the data did not meet the assumptions of parametric tests. Larvae growth parameters were analysed using SPSS software (IBM Corp, NY, USA). Texture measurements were carried out in three technical replicates. The statistical evaluation of the force values at 15 mm penetration was carried out also using ANOVA (*p* < 0.05) with a subsequent Tukey post hoc test using OriginPro 9G software (OrginLab, Northampton, MA, USA).

## 3. Results

### 3.1. Experiment 1: The Influence of Different Fibre Components and Nutrient Levels on BSFL Growth

**Figure 1:**
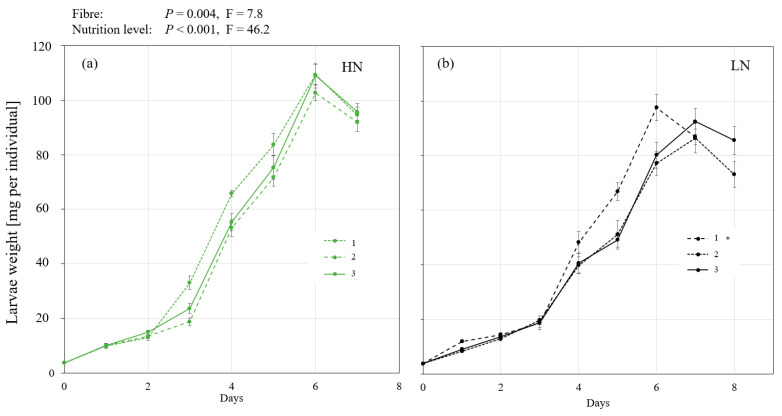
The growth of black soldier fly larvae (BSFL) on different fibre components and two nutrient levels (experiment 1). BSFL were grown in high-nutrient (HN, (**a**)) or low-nutrient (LN, (**b**)) substrates and with 3 fibre components (1: powder cellulose, 2: lignocellulose, or 3: ground straw). The single-larva fresh weight was measured over time, and the daily means and standard errors are shown in the graph. Linear mixed-effect model analysis followed by a Bonferroni post hoc test revealed a significant impact of both factors (nutrient level and fibre) on growth over time. The significant difference between fibre treatments in a nutrient level is indicated by an asterisk.

The growth of larvae reared in different nutrient levels and different fibre components were measured and is shown in Figure 1. The larvae reached their maximum weight on day 6 or 7 depending on the treatment, and the two factors (nutrient level and fibre) significantly influenced the growth of BSFL over time (*P* and F values are shown in Figure 1). BSFL had improved growth on the high-nutrient (HN) substrate. In the low-nutrient (LN) substrate, BSFL grown on the cellulose-supplied substrate (treatment 1) reached their maximum weight one day earlier than those grown on the lignocellulose or the straw substrates (2 and 3, respectively), and the difference between the treatments was significant.

**Figure 2:**
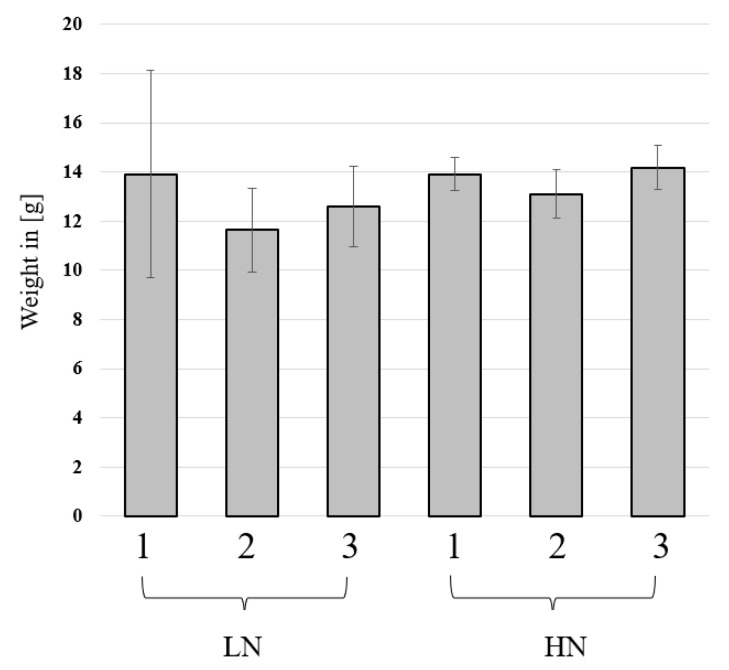
The dry yield of black soldier fly larvae (BSFL) grown on different fibre components and two nutrient levels. BSFL were grown in high-nutrient (HN) or low-nutrient (LN) substrates and with 3 fibre components (1: powder cellulose, 2: lignocellulose, or 3: ground straw). The larvae were harvested and dried, and the final dry yield was measured. The mean is shown with the standard deviations. Kruskal–Wallis test (n = 5, *p* < 0.05) revealed no effects on the dry yield.

The nutrient level had a significant impact on the fresh yield (data not shown) which was significantly lower in the low-nutrient (LN) treatment. The fibre component did not influence the final fresh and dry yields, and only a trend was observed (Figure 2).

**Figure 3:**
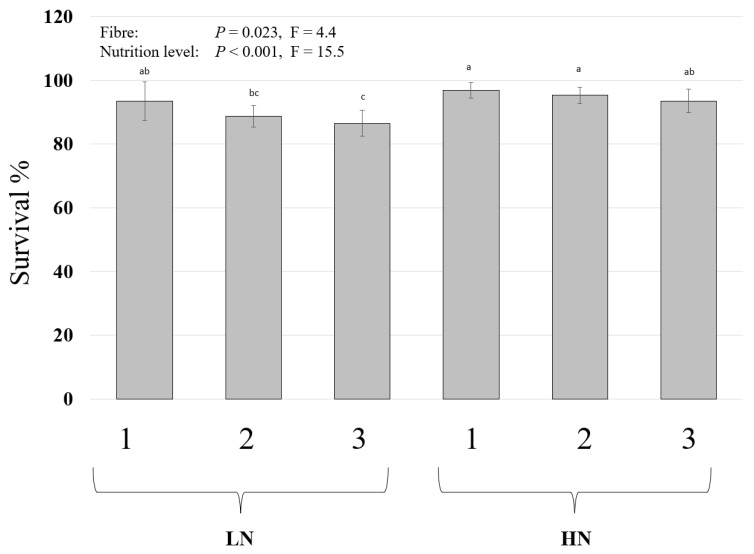
The survival of black soldier fly (BSF) larvae grown on different fibre components and two nutrient levels. BSFL were grown in high-nutrient (HN) or low-nutrient (LN) substrates and with 3 fibre components (1: powder cellulose, 2: lignocellulose, or 3: ground straw). The larvae were harvested, and the survival was assessed. Two-way ANOVA (n = 5, *p* < 0.05) followed by Bonferroni post hoc test revealed that both factors influenced the survival of the larvae without an interaction between the factors. Significant differences are found between treatments 1 and 3 (main effect), and pairwise differences are indicated by different letters above the column.

Both factors influenced the survival of the larvae, and the values ranged between 86% and 96% (Figure 3). The survival was higher in the substrates with higher nutrient level (HN) in comparison to the substrates with the lower nutrient level (LN) (main effect). The BSFL had significantly higher survival in the cellulose-supplied substrates (treatment 1) in comparison to the straw-supplied substrates (treatment 3). The feed conversion ratio (FCR) ranged between 5.5 and 4.2 and was influenced by the nutrient level but not the fibre component (data not shown).

**Figure 4:**
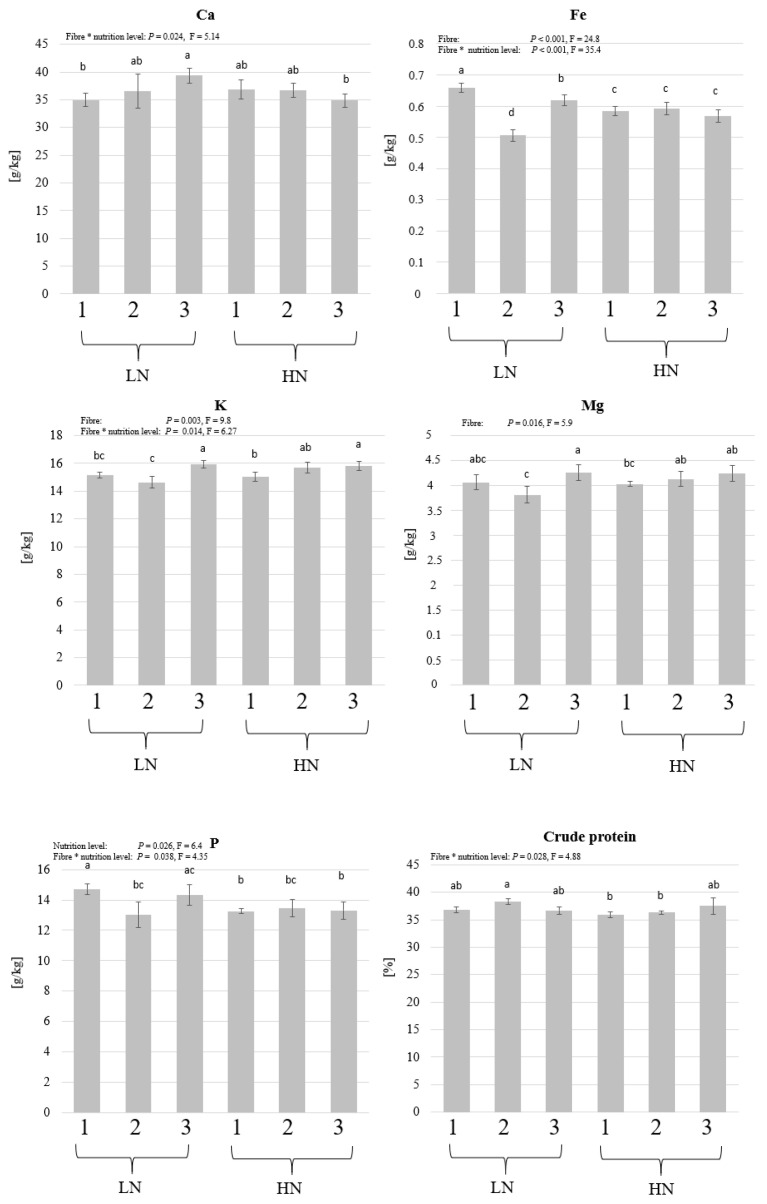
Protein and micronutrients in black soldier fly (BSF) larvae grown on different fibre components and two nutrient levels. BSFL were grown in high-nutrient (HN) or low-nutrient (LN) substrates and with 3 fibre components (1: powder cellulose, 2: lignocellulose, or 3: ground straw). Shown are the means and standard deviations of crude protein, Ca, Fe, K, Mg, and P in the harvested larvae. Two-way ANOVA (n = 5, *p* < 0.05) followed by a Bonferroni post hoc test revealed a significant impact of the factor “fibre” on Fe, K, and Mg, and an influence of the “nutrient level” only on the P level. An interaction was observed in Ca, Fe, P, K, and crude protein concentrations. The letters above the columns indicate the different groups and the asterisk (*) between factors indicates the interaction.

The protein concentration and the concentrations of Ca, Fe, K, Mg, and P were analysed and compared between the treatments. The two factors did not influence the concentration of proteins in the larvae, but an interaction was observed (*p* = 0.028, F = 4.88) as the protein concentration is higher in the low nutrient level (LN) for the lignocellulose-supplied substrate (2) (Figure 4). An interaction, with the absence of a single factor effect, was observed in the concentration of Ca, which increased in the low nutrient level (LN) in the straw-supplied substrates (3) (*p* = 0.024, F = 5.14). An interaction between the factors was also observed in the concentration of Fe (*p* < 0.001, F = 35.4) and K (*p* = 0.014, F = 6.2). The factor “fibre” influenced the concentrations of Fe (*p* < 0.001, F = 24.8), K (*p* = 0.003, F = 9.8), and Mg (*p* = 0.016, F = 5.9), as the lignocellulose-supplied substrate (2) led to a lower concentration of these elements. The concentration of P was higher in the low-nutrient treatments (LN), and an interaction between the two factors was observed.

### 3.2. The Influence of Different Straw Particle Sizes

**Figure 5:**
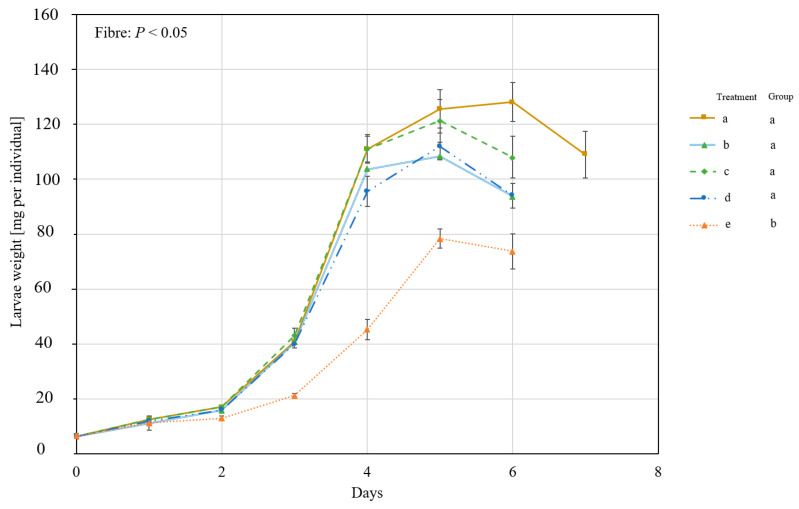
The growth of BSFL on substrates with different straw particle sizes (experiment 2). BSFL were grown on substrates that contain >3 mm straw (a), 2–3 mm straw (b), 1.25–2 mm straw (c), 1–1.25 mm straw (d), or ≤1 mm straw (e). The single-larva fresh weight was measured over time, and the daily means and the standard errors are shown in the graph. Linear mixed-effect model analysis followed by a Bonferroni post hoc test revealed significant differences between the treatments. Different groups are indicated by the different letters beside the legends.

The size of straw particles influenced the growth of the larvae as substrates with a lower particle size led to lower growth over time (Figure 5). The difference in the growth reflected also on the final yield produced per box as the lowest fresh and dry yields were recorded for the smallest particle size “e” (*p* = 0.012, F = 4.2 for the fresh yield, and *p* = 0.012, F = 6.8 for the dry yield). The values were 26 g (SD = 6.2) and 8 g (SD = 1.8) for the fresh yield and the dry yield, respectively. The highest fresh and dry yields were recorded in the treatment with the biggest straw particle size “a” and were 42 g (SD = 10.9) and 15 g (SD = 3.2), respectively (data not shown).

Additionally, a difference in the survival of larvae was observed (*p* = 0.009, F = 4.5) and ranged from 96% (SD = 2.1) to 84% (SD = 6.3). The survival also decreased in the treatments with the lowest particle size (data not shown). The feed conversion ratio (FCR) also differed between the treatments (*p* = 0.003, F = 5.8), and the lowest particle size led to the highest FCR value (12.2). The FCR values in the rest of the treatments ranged between 7.1 to 8.2 and did not differ significantly (data not shown).

**Figure 6:**
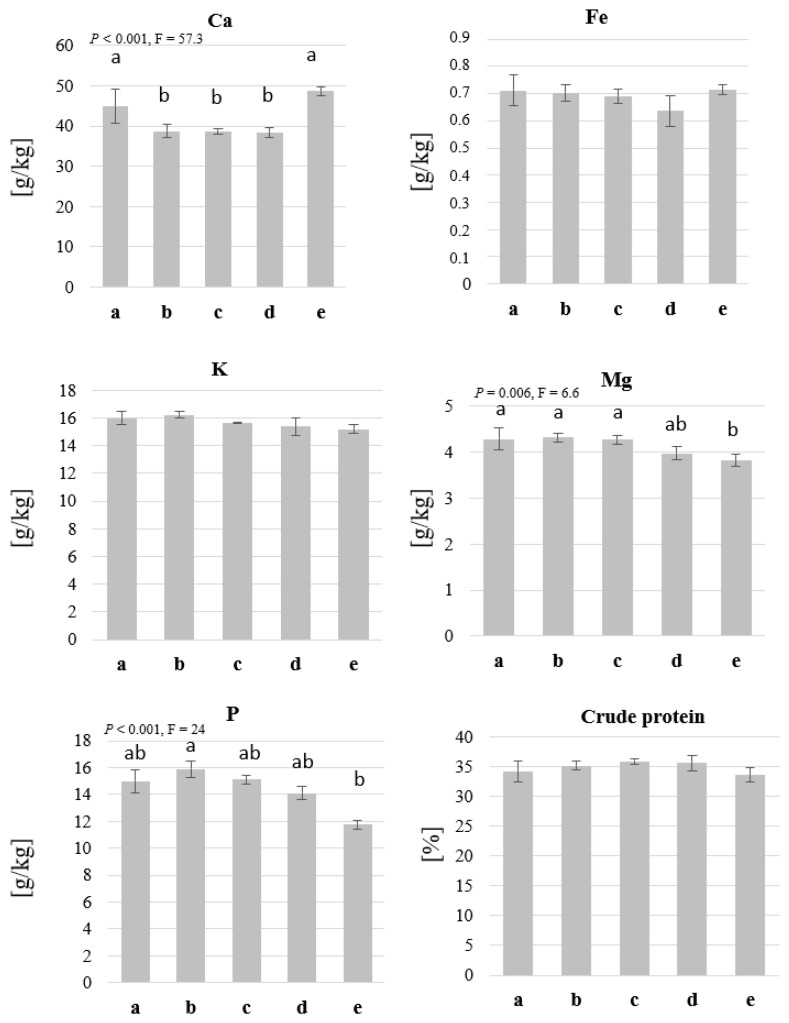
Protein and micronutrients in BSFL grown on substrates with different straw particle sizes. BSFL were grown on substrates that contain >3 mm straw (a), 2–3 mm straw (b), 1.25–2 mm straw (c), 1–1.25 mm straw (d), or ≤ 1 mm straw (e). The larvae were harvested and analysed for crude protein, Ca, Fe, K, Mg, and P concentrations. ANOVA (n = 5, *p* < 0.05) followed by a Bonferroni post hoc test revealed significant differences between the treatments in Ca, Mg, and P concentrations, and no differences in the case of Fe, K, and crude protein. The means and standard deviations are shown in the graph, and the letters above the columns indicate the different groups.

In this experiment, the concentrations of Ca, Fe, K, Mg, P, and protein were also analysed. No differences between the treatments were observed in the case of Fe, K, and proteins. However, the concentration of Ca was higher (*p* < 0.001, F = 14) in treatments with the lowest and the highest straw particle size (treatments a and e). The substrate with the lowest straw particle size led to the lowest concentrations of Mg and P in the produced BSFL (Figure 6).

## 4. Discussion

It has been shown that factors such as substrate nutritional composition and substrate moisture can have a big impact on BSFL growth and composition [32,33,34]. Substrate moisture has been investigated in numerous studies. For example, Bekker et al. [24] found that a substrate water content of 45 or 65% leads to lower mortality in BSFL grown on chicken feed in comparison to 75% moisture. In other studies, when substrates with high fibre content have been used, 40% substrate moisture seemed to cause 100% mortality whereas the larvae performed better at 50–70% moisture [25,35].

Water occurring in moist substrates (or any organic material) can be free or bound water [36], and the status in which the water occurs influences its bioavailability and numerous physical properties in the material [37]. It is, therefore, important to take the status of water into consideration and explore how physical parameters in substrates can influence the rearing process.

Substrate moisture also depends on many factors in the BSFL bioconversion process. Efficient ventilation, for example, can be an approach to enhance evaporation and reduce excess substrate moisture [28], improving bioconversion and enabling easier larvae separation at the time of harvest. Additionally, growing larvae in higher densities and/or bigger containers increases the temperature in the substrate [18], which could also enhance evaporation.

Aside from ambient conditions, substrates that differ in chemical composition and/or water content possess different textures and physical properties which could, consequently, influence the performance of BSFL. This study, therefore, compared the growth and chemical composition of BSFL cultivated on substrates that differ in physical properties with similar water contents. To develop substrates that mainly vary in physical properties, three different types of fibre additives were used (shown in Table 1). The fibres were, namely, purified cellulose and lignocellulose, and commercial wheat straw which normally contains cellulose (28–39%), lignin (16–25%) hemicelluloses (23–24%), and small amounts of ash and proteins [38]. The three fibre additives were compared at two nutritional levels in a factorial experiment (Table 1). A second experiment was carried out only by using straw with different particle sizes prepared from the same batch to neglect the marginal variances in mineral composition and nutritional values of the added fibres (Table 1). Furthermore, the bioavailability of fibres could differ as suggested in other studies [39,40].

The results of both experiments show that altering the physical properties through the fibre component in the substrate can influence the growth of BSFL as shown in Figure 1 and Figure 5. In the first experiment (Figure 1), the effect was more pronounced in the low nutrient (LN) treatments which contain higher amounts of fibres and fewer nutrients compared to the high nutrient (HN) treatments (Table 1). Understanding whether the effect observed is related to the nutritional value of the feed or to the proportion of fibres added is a topic for future research.

The tested substrates required a different force per distance in the texture measurement (Table 2) and had different bulk densities (Table 1), which is a measurement of weight per unit volume of the material. The higher the bulk density, the lower is the pore volume in the substrate. The texture measurement gives insights into the mechanical characteristics of the material [41]. In the case of the BSFL bioconversion process, the larvae continuously move and explore the substrate, and this activity, in addition to feeding, which is mechanically facilitated by the buccal apparatus [42], might be hindered when the substrates exhibit high mechanical resistance or when the substrate has a low pore volume. We, therefore, hypothesised that a relation might be observed between the growth of larvae over time and the obtained force per distance values, and/or the bulk density of wet substrates at the start of the experiment. Nevertheless, this was not observed as the cellulose (1) and the straw (3) treatments had higher insect growth over time (Figure 1) in comparison to the lignocellulose (2) which had lower substrate bulk density at the start of the experiment in comparison to the straw treatment (3). However, the growth of the larvae in both experiments seems to negatively correlate with the dry bulk density of the fibre component (Table 1 and Table 3) added to the substrate. The lower bulk density of the fibre component means higher pore volume as the substrate dries during consumption, which could ultimately allow better aeration and most likely the free movement of the larvae. BSFL have been shown to drown in low-porosity and high-moisture substrates [28]. Gligorescu et al. [43] have suggested adding chicken feed to wastes that have higher moisture than 85% and indicated that this confers benefits to the physical properties of the substrate and increases porosity. Additionally, Shumo et al. [44] compared the growth of BSFL on substrates such as cow dung and spent grains, and mentioned that the “thick” texture of dung might have also contributed to the lower larvae performance as it might have limited larvae mobility and access to nutrients. These indications in the literature, however, have not been tested in an experimental setup to assess the impact of these characteristics of the substrates, and this was the aim of this study.

In addition to the mechanical direct effect, aeration has been shown to increase the growth of larvae [25]. Enhanced oxygen supply can be facilitated by higher porosity, thus leading to the improved growth of BSFL. Producers use active ventilation to improve aeration and enhance gas exchange [28], but this could lead also to the fast drying of the substrates. Optimising the structure and the porosity of the substrates by using a fibre component with optimal bulk density and water holding capacity can be a way to improve oxygen diffusion and minimise the occurrence of anaerobic conditions in the substrate.

In addition to growth over time, the final yield and FCR values obtained from the second experiment differed between the treatments. The lower maximum BSFL weight achieved (Treatment e, Figure 5) could indicate that the nutrients in these treatments were less available for the larvae despite having the same amount of chicken feed and straw in the start substrate. The factors that influence nutrient availability in relation to fibre properties and particle size must be further investigated.

Besides growth, the survival of the larvae was influenced by the nutrient level and the fibres in both experiments. The survival of BSFL has been shown to be influenced by the substrate nutrient composition [35,45], which was also observed in this study where the survival decreased when larvae had fewer nutrients (Figure 3). Other factors have been shown to also influence BSFL survival such as temperature, and the size of the rearing box [18]. The survival values obtained in this study (85% to 96%) were relatively high in comparison to other studies that used agricultural by-products such as winery by-products (75%) [32] and meat meal [46]. It is, however, known that unbalanced diets, even those high in proteins, could lead to high mortality [27]. The chicken feed used in this study can be considered a balanced diet which can explain the relatively high survival observed in this study.

Lalander et al. [28] have found a negative correlation between substrate water content and BSFL survival. The authors mentioned that high water content can negatively affect substrate structure and porosity, and they observed a separation of water from the rest of the substrate. Our study suggests that the water holding capacity of the fibres added to the substrates could improve the structure and reduce the mortality of the BSFL; this effect may potentially occur also in the case of substrates with high water contents. As shown in Table 1 and the mortality values, the survival was the highest when fibre with higher water holding capacity is added; this also led to a substrate structure that prevented the “sinking” of larvae in the separated/free water. In the substrates tested in this study, the separation of liquids was observed in the straw variant (treatment 3) in the first experiment and was also higher in the straw variance “e” in the second experiment. Testing whether improving the physical properties of suboptimal substrates (e.g., high moisture and unbalanced nutrient composition) reduces the mortality of BSFL could be a topic for future research.

The chemical analysis results revealed that the nutritional composition of BSFL could be altered by changing the fibres used in the substrate. The crude protein concentration in the first experiment was not influenced by a single factor, but an interaction was observed between the fibre and nutrition levels (Figure 4). Protein concentration could be influenced by the diet [47] and by other factors such as the larval density in the rearing box, which could influence the availability of nutrients for a single larva [18]. In the presented study, the LN-2 larvae had higher protein concentration than the HN-2 larvae, which, in this case, cannot be explained by nutrients availability but is likely due to the dilution of proteins in the significantly higher larval biomass, or the different developmental stage of the larvae. This may also explain the higher P levels in the LN treatments as P has been shown to be more abundant in smaller larvae even when the initial substrate had higher P contents [31]. Generally, the presence of several elements in the larval biomass can be affected by the diet [48]. Fe and K concentrations in the BSFL biomass were influenced by the fibre component in the first experiment (Figure 4) but not in the second (Figure 6). The values of Fe differed only among the low nutrition (LN) treatments and, unlike K, did not increase based on the Fe concentration in the fibre (which is the highest in straw “fibre 3”). The concentration of Mg was also influenced by the fibre used, which seems to be related to the substrate as it, in addition to K, occurs in commercial wheat straw (Table 1) in a higher concentration than the purified cellulose and lignocellulose used in treatments 1 and 2.

Ca and Mg are known to accumulate in the BSFL, and these two elements are of high importance in feedstuff [18,49,50]. The concentration of Ca and Mg has been shown to differ up to more than 6- and 4-fold, respectively, based on the diet and larval size, and the smaller larvae seemed to have higher concentrations of these two elements [47]. In the second experiment, the larvae grew more in treatment “a” and accumulated more Ca, which might be due to the Ca biomineralisation as the larvae develop and build thicker chitin [51]. Additionally, the different harvest time-points in some treatments (based on BSFL growth) could lead to the differential accumulation of elements in relation to larvae development. Additionally, we assume that the water availability, which is influenced by the water holding capacity of the fibres used, and the differential evaporation from the substrates, could have resulted in the different availability of these elements in their water-soluble forms. Nevertheless, the accumulation of elements in the larvae in relation to development and growth needs further investigation.

## 5. Conclusions

This study aimed to give insights into the effect of substrates’ physical properties on larvae growth over time and the nutrient composition of produced larvae. As shown in the results, the growth and survival of BSFL can be affected when using different fibres or by changing the particle size of the fibre component. Larvae that grow on substrates supplied with different fibres can have different concentrations of K, Mg, Ca, and Fe. Altering the particle size of straw in the substrate can influence P, Mg, and Ca concentrations.

## 6. Outlook and Future Remarks

This study did not aim to identify optimal substrate physical properties but can be the first brick towards building knowledge about the influence of their important and neglected physical properties. The penetration test used to assess the texture of the initial substrate did not reveal any relation to larvae growth. However, this analysis is only one of many methods to describe the physical properties of substrates. Texture analyses could be complemented with viscosity measurements using a suitable method that considers challenges such as the expulsion of water from the substrate and the wide range of consistencies between the different substrates.

However, measuring the amounts of free and bound water in the substrates was not conducted in this study, but the relation between the occurrence of free water and larval mortality can be a topic for future research and it may enable the definition of values that allow maximum larval survival. We suggest that the optimisation of BSFL-rearing substrates should include both improving their nutritional values and adjusting their physical properties. Other factors that interfere with aeration should also be considered in future research such as the evaporation from the substrate which could influence the availability of nutrients, the microbiome, and ultimately BSFL growth.

## Figures and Tables

**Table 1:** Treatments and feed mixtures in the first experiment (wet basis). The characteristics and partial composition of the dry fibre components are provided under the table.

Treatments		Chicken Feed [%]	Water [%]	Powder Cellulose (%)	Concentrated Lignocellulose [%]	Blended Straw [%]	Bulk Densities of the Final Wet Substrate [g L^−1^]
Factor 1	Factor 2						
1	High nutrition (HN)	20.33	73.17	6.50	-	-	635
2		20.33	73.17	-	6.50	-	720
3		20.33	73.17	-	-	6.50	836
1	Low nutrition (LN)	14.63	73.17	6.50	-	5.70	814
2		14.63	73.17	-	6.50	5.70	1032
3		14.63	73.17	-	-	12.20	1072

Powder cellulose: Average fibre length = 700 µm; water holding capacity = 9.45 mL g^−1^; dry bulk density = 118 [g L^−1^]; wet bulk density = 1116 [g L^−1^]; N = 0.035 %; Ca = 0.84 [g kg^−1^ DM]; Fe = 18.93 [mg kg^−1^ DM]; K = 38.97 [mg kg^−1^ DM]; Mg = 221.5 [mg kg^−1^ DM]; P = 8.17 [mg kg^−1^ DM]. Concentrated lignocellulose: average fibre length = 280 µm; water holding capacity = 5.5 mL g^−1^; dry bulk density = 424 [g L^−1^]; wet bulk density 1711 [g L^−1^]. N = 0.034%; Ca = 1.03 [g kg^−1^ DM]; Fe = 19.53 [mg kg^−1^ DM]; K = 114.98 [mg kg^−1^ DM]; Mg = 174.47 [mg kg^−1^ DM]; P = 9.11 [mg kg^−1^ DM]. Blended straw: average fibre length = 1.25 mm; water holding capacity = 3.6 mL g^−1^; dry bulk density = 183 [g L^−1^]; wet bulk density = 1219 [g L^−1^]. N = 0.64 %; Ca = 3.31 [g kg^−1^ DM]; Fe = 319.9 [mg kg^−1^ DM]; K= 13.6 [g kg^−1^ DM]; Mg = 1.15 [g kg^−1^ DM]; P = 1.27 [g kg^−1^ DM].

**Table 2:** The force needed for the penetration of the different substrates used in the first experiment for over 15 mm. The slopes of mean values of force over distance curves and the corresponding coefficients of determination (R^2^) are shown. The presented values are the mean of three measurements (technical replicates) ± the standard deviations. Different letters indicate significant differences between samples.

Treatment		F_15mm_ [N]	Slope [N/mm]	R^2^_slope_
Low nutrition (LN)	1	2.44 ^a^ ± 0.44	0.162	0.9911
	2	1.24 ^a,b^ ± 0.39	0.084	0.9997
	3	0.29 ^b^ ± 0.02	0.019	0.9996
High nutrition (HN)	1	6.63 ^c^ ± 0.82	0.457	0.9993
	2	4.68 ^d^ ± 0.58	0.342	0.9952
	3	2.07 ^a^ ± 0.33	0.145	0.9975

**Table 3:** Properties of straw fractions used to prepare the substrates.

Treatment	Particle Size [mm]	WatHC [mL g^−1^]	DryBDens [g L^−1^]	WetBDens [g L^−1^]
a	3–15	6.46	83.2	762.8
b	2–3	6.38	110.5	1031
c	1.25–2	3.7	165.7	1179.9
d	1–1.25	3.68	312.1	1223.7
e	≤1	3.16	442.1	1494.3

WatHC: water holding capacity, DryBDens: dry bulk density, WetBDens: wet bulk density. (a–e): names/abbreviations of the treatments, and its indicated in the header that these are the different treatments).

## Data Availability

The data presented in this study are available on request from the corresponding author.
